# Otosclerosis Associated with a De Novo Mutation −832G > A in the TGFB1 Gene Promoter Causing a Decreased Expression Level

**DOI:** 10.1038/srep29572

**Published:** 2016-07-11

**Authors:** Saurabh Priyadarshi, Kirtal Hansdah, Chinmay Sundar Ray, Narayan Chandra Biswal, Puppala Venkat Ramchander

**Affiliations:** 1 Institute of Life Sciences, Nalco Square, Chandrasekharpur, Bhubaneswar, India; 2 Department of Ear, Nose, and Throat (ENT), Shrirama Chandra Bhanj (SCB) Medical College, Cuttack, India

## Abstract

Otosclerosis (OTSC) is defined by abnormal bone remodeling in the otic capsule of middle ear which leads to conductive hearing loss. In our previous study, we have identified a de novo heterozygous mutation −832G > A in the promoter of TGFB1 in an otosclerosis patient. In the present study, we progressively screened this mutation in a cohort of 254 cases and 262 controls. The family members of the patient positive for −832G > A variation were also screened and found inheritance of this variation only to her daughter. Interestingly, this variation is associated with a decreased level of the TGFB1 transcript in the patient compared to her parents and controls. *In silico* analysis of this mutation predicted the altered binding of two transcription factors v-Myb and MZF1 in the mutated promoter sequence. Further, functional analysis of this mutation using *in vitro* luciferase and electrophoretic mobility shift assays revealed that this variation is associated with decreased gene expression. In conclusion, this study established the fact that TGFB1 mutation −832G > A altered the TGFB1 promoter activity, which could affect the susceptibility to otosclerosis development. Further, systemic analysis of TGFB1 gene sequence and expression analysis of this gene might reveal its precise role in the pathogenesis of otosclerosis.

Otosclerosis (OTSC) is characterized by an abnormal bone remodeling in the otic capsule which leads to stapedial fixation resulting in conductive hearing loss. Otosclerosis is divided in to histological and clinical types. Clinical otosclerosis is the major cause of acquired hearing loss with a prevalence of 0.3–0.4%. Histological otosclerosis occurs most frequently without clinical symptoms^1^. Etiopathogenesis of otosclerosis is yet unexplained, however, genetic predisposition, disturbed bone metabolism, persistent measles virus infection, autoimmunity, hormonal and environmental factors may play contributing roles in the pathogenesis of otosclerosis^2^. Genetic predisposition for disease has been recognized after the identification of ten monogenic loci (OTSC1 - OTSC5, OTSC7, OTSC8 and OTSC10); however, the causative genes within these regions are undefined till date^3^. Sporadic cases are common and genetic association studies have shown the association of COL1A1, TGFB1, BMP2, BMP4 and RELN gene with otosclerosis^3^. Common variants in TGFB1 gene with functional significance were found to be significantly associated with otosclerosis in different ethnic population^3^,^4^. However, rare variants in the TGFB1 gene with pathogenic effects are very rarely found in otosclerosis cases^5^. It has been reported that low frequency rare genetic variants can have a large impact in the etiology of complex traits^6^. Recently, we have shown the genetic association of −509C > T SNP in TGFB1 gene towards the susceptibility of otosclerosis development and also identified a de novo heterozygous mutation −832G > A in the promoter region of TGFB1 in one patient with otosclerosis^3^. TGFB1 promoter region variants have functional relevance that likely impacts the pathogenesis of numerous TGFB1 related disorders^7^. To determine whether this is true for −832G > A variation in the etiology of otosclerosis, we expanded the screening of this variation and characterized to assess the role of this variation in relation to the disease.

## Results

### Clinical analysis

**Figure 1:**
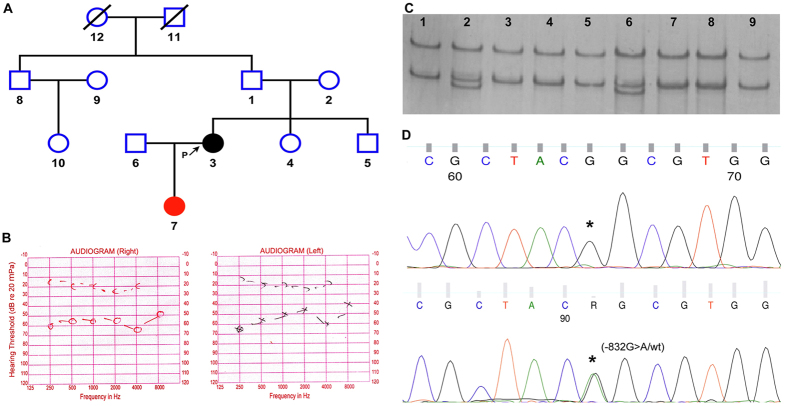
(**A**) Pedigree of the family showing the affected proband (circle 3) and her 1.5 year old daughter with uncertain disease status (circle 7). (**B**) On audiometric assessment, pure tone audiometry showed a bilateral conductive hearing loss in proband with an extent of moderate hearing loss 55 dB in right ear and 50 dB in left ear. (**C**) PCR based SSCP analysis of individuals from the pedigree showing the heterozygous banding pattern for −832G > A mutation in the proband and her 1.5 year old daughter (lane 2 & 6), the proband parents and other relatives were normal for this mutation (lane 1, 3–5 & 7–9). (**D**) This sequence variation was validated by direct sequencing in both forward and reverse direction. The chromatograms are showing the homozygous ‘GG’ and heterozygous ‘GA’ genotypes for −832G > A mutation in the promoter of TGFB1 gene.

In the present report, we have described a 1-bp substitution in the promoter region sequence of TGFB1 in a 25 year female patient with bilateral moderate conductive hearing loss. She was born at full term to unrelated parents who did not have any symptoms of hearing impairment (Fig. 1A). At the age of 19, the proband showed mild symptoms of otosclerosis. On follow up slowly the hearing condition worsens from mild to moderate. Otoscopic examination of the patient showed normal tympanic membranes. Tuning fork test showed Rinne negative bilaterally and Weber test was localized to the centre. On audiometric assessment, pure tone audiometry showed a bilateral conductive hearing loss (air bone gap 30–35 dB) with an extent of moderate hearing loss (Fig. 1B). Impedance audiometry showed As’ type tympanogram with absence of ipsilateral and contralateral stapedial reflexes. Radiologic assessment showed a hypo dense bony lesion around the stapes footplate. The patients father, mother, brother, sister and other relatives have normal hearing condition upon audiometric assessment.

### Identification of de novo mutation in TGFB1 promoter

SSCP analysis of promoter region of TGFB1 identified a de novo 1 base pair (−832G> A) substitution located 1670 bp upstream from the transcription start site (NM_000660.5) in a patient with otosclerosis (Fig. 1C). This sequence variation was validated by direct sequencing in both forward and reverse direction (Fig. 1D). The mutation was positive on the patient and her 1.5 year old daughter only; however, her parents and other relatives were negative for this variation. This mutation was suggested de novo because it was not detected in DNA isolated from patient’s parents as well as in any other DNA samples from 253 cases and 262 healthy individuals used as controls.

### Expression analysis of TGFB1

**Figure 2:**
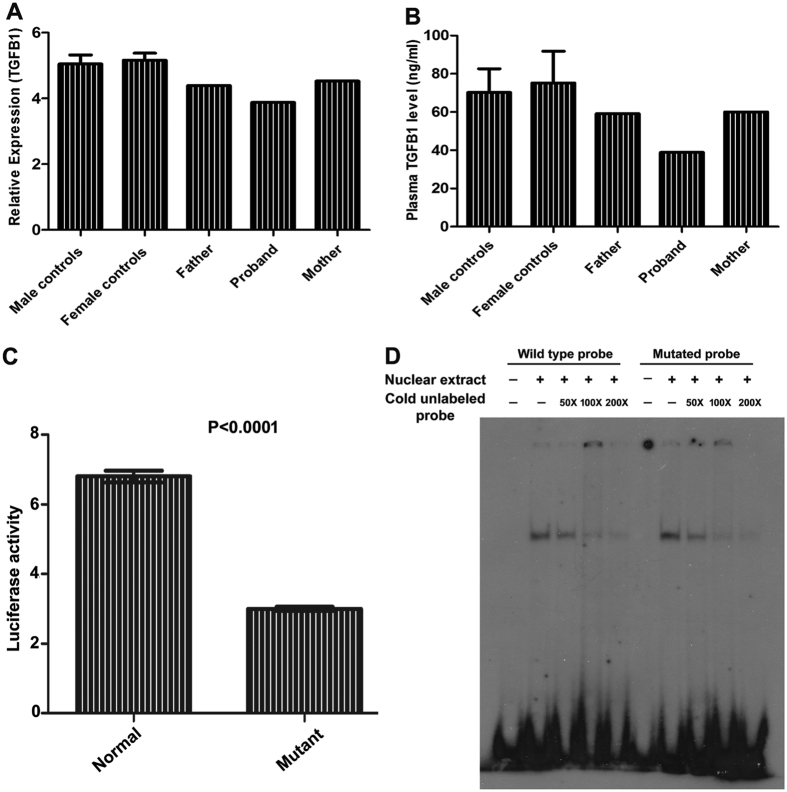
(**A**) TGFB1 gene expression levels were quantified in triplicates in patient, her parents and controls (6 males; 6 females) using Real time quantitative PCR. We found a decreased TGFB1 transcript level in patient when compared to the parents and control subjects. (**B**) Blood plasma level of TGFB1 was estimated in triplicates in patient, her parents and controls (6 males; 6 females) using enzyme linked immunosorbent assay (ELISA). The TGFB1 plasma level was found to be decreased in the patient when compared to her parents and control subjects. (**C**) The wild type and mutated human TGFB1 promoter (1199 bp) was cloned into the SacI and HindIII sites of pGL3 basic promoter less vector which contained the firefly luciferase gene. After transient transfection in to human embryonic kidney (HEK) 293 cells, the luciferase assays were performed with dual-glo luciferase assay system, and the results gave a significant decrease (2.2 fold, P < 0.0001) of the reporter gene expression under the control of mutated TGFB1 promoter. Results were expressed as F-luc/R-luc means ± SEM. (**D**) Electromobility shift assay (EMSA) was performed on nuclear proteins extracted from human MCF7 cells with either wild (lanes 1–5) or mutant (lanes 6–10) type probes with & without 50X, 100X and 200X competitors. DNA-protein complex migration pattern was detected in both wild type and mutated probes.

To test whether this mutation could alter the TGFB1 transcript level, we performed a real time quantitative PCR on total RNA extracted from fresh blood of the patient, her father, mother and control subjects (six male and six female). We identified a decreased level of TGFB1 transcript level in the patient compared to her parents and control subjects (Fig. 2A). Furthermore, we evaluated the TGFB1 level in the blood plasma of patient compared to her parents and controls subjects. Interestingly, we identified an approximately 1.54 fold decrease in the TGFB1 blood plasma level compared to her parents and 1.8 to 1.9 fold decrease in plasma level compared to the male and female control subjects (Fig. 2B).

### Functional characterization of de novo mutation

To check the impact of this variation on TGFB1 expression, we performed *in vitro* transcription assays and found a significant decrease (2.2 fold, P < 0.0001) of the luciferase gene expression under the control of the mutated TGFB1 promoter (Fig. 2C). This functional analysis suggests that the mutation leads to decreased luciferase activity. We next wondered whether the reduce luciferase activity caused by the nucleotide substitution was due to alteration in transcription factor (TF) binding sites located in this region. We searched for potential modification involving TF binding sites on the promoter region with TFSEARCH prediction software. The comparative analysis of normal and variant sequences indicated the substitution would be associated with the gain of altered binding of two transcription factors v-Myb and MZF1. To investigate whether the mutant allele of −832G > A mutation would modify the binding affinity to nuclear protein, we performed EMSA with MCF7 cells nuclear protein extracts with double stranded oligonucleotide probes containing either allele. EMSA revealed the possible binding of any of the two transcription factors in both the wild type and mutated promoter sequences (Fig. 2D). Both normal and mutated alleles were examined by EMSA, but we were not able to differentiate the nuclear protein binding between the two transcription factors.

The obtained results from quantitative real time PCR, ELISA, luciferase and EMSA assay experiments indicate that the nucleotide variation might be the cause of decreased TGFB1 expression.

## Discussion

TGFB1 is a multi factorial growth factor that regulates a broad range of biological process. Based on the genetic association of TGFB1 SNPs and its differential expression in otosclerotic tissues, we and other researchers believe that TGFB1 is a possible candidate gene in the etiopathogenesis of otosclerosis^3^,^4^,^8^.

Although the “common disease common variant” hypothesis is true for many complex diseases^6^, there are evidences that rare variants can have large effect on the phenotypes as well^9^. Rare variants in the TGFB1 gene with possible functional significance have been found in otosclerosis cases in some ethnic populations^5^. In this study, we reported the description of a de novo mutation −832G > A in the promoter region of TGFB1 gene inheriting from an otosclerosis patient to her daughter. Promoter region of TGFB1 gene consist of an enhancer like regulatory element (−1132 to −731) and two strong negatively regulatory regions (−1362 to −1132 and −731 to −453). Both the negative regulatory region and enhancer like region regulate the cell specific expression of human TGFB1^10^. TGFB1 promoter region variants −509C > T and −800G > A have functional relevance that likely impacts the pathogenesis of numerous TGFB1 related disease^7^. However, nothing is known regarding the altered expression of TGFB1 that would be caused by the −832G > A mutation. We investigated this issue by expanding the screening of this mutation in an extended family and other cases and controls. This mutation was suggested as rare de novo variation because it was detected in only one patient and in her daughter and was absent in other DNA samples. Out of the two major approaches for identification of candidate genes in complex disorders: linkage analysis and association studies, none of them is able to detect susceptibility loci that harbour rare but deleterious variations. However, it is believed that some common disease may be caused by rare variants as majority of them are deleterious and are of significance in disease manifestation^11^.

This variant is associated with decreased TGFB1 expression in the patient compared to her parents and control subjects. The variable TGFB1 expression among the male and female control subjects, suggests a complex regulation of TGFB1 expression. TGFB1 controls both osteoblast and osteoclast differentiation, and therefore balances bone formation and resorption. TGFB1 regulates the expression and secretion of OPG and RANKL. In our previous study, we have reported an alteration in OPG and RANKL mRNA expression in the stapes tissues of otosclerotic patients^12^. It has also been found that decreased TGFB1 level leads to increased Th17 cell population which results in increased TNFα production^13^. The increased TNFα expression as a consequence of measles virus induced inflammatory reaction has been found in the human otosclerotic tissues^14^. It is possible that decreased TGFB1 expression have impact on these molecules, however, additional studies are needed on this light to understand the complete TGFB1 signaling mechanism in otosclerosis development.

Interestingly, *in silico* analysis for this variation predicted the altered binding of two transcription factors v-Myb and MZF1 in the mutated promoter sequence. EMSA revealed the possible binding of any of the two transcription factors in both wild type and mutated promoter sequences. Both the transcription factors are transcriptional regulators however; their role in tumorigenesis is antagonistic in hemopoietic compartment^15^,^16^. v-Myb is a mutated form of c.Myb which plays central role in the regulation of hematopoiesis and tumorigenesis including the regulation of miR-155 in B-cells^16^. A recent report has described the OPN-MZF1-TGFB1 linked pathway and has shown the effect of MZF1 on the TGFB1 promoter activation and synthesis^17^. Our functional analysis showed a very significant and consistent difference in luciferase activity between the wild type and mutated promoter sequence with mutated allele ‘A’ as inactive variant. However, some variants have been shown to influence reporter gene activity without allele specific difference in DNA protein interaction. Changes in EMSA condition might effects DNA protein interaction which was not apparent under the experimental conditions used here^18^. Although, this variant was found to be significantly associated with decreased gene expression, however, we acknowledge the limitation of this study because of the presence of only one causative variation in the population of 254 otosclerosis cases and the lack of functional evidences on bone structures from the patient carrying the mutation.

In summary, our results indicate that down regulation of TGFB1 at transcript and protein level due to de novo mutation −832G > A is associated with otosclerosis development. The results from this study bring a new insight regarding the phenotypic spectrum of TGFB1 mutations and suggest that the systemic analysis of TGFB1 gene sequence and expression analysis of this gene might reveal more individuals with mutations.

## Materials and Methods

### Study Subjects

The proband, a twenty five (25) year old female presented with a 6 years history of progressively worsening hearing loss to the Department of Ear, Nose and Throat (ENT), SCB Medical College, Cuttack, Odisha. The patient was diagnosed on the basis of otoscopy, tuning fork test (TFT), pure tone audiometry (PTA), impedance testing and high resolution computer tomography (HRCT) scanning. There were no associated complaints of otorrohoea, tinnitus, vertigo/imbalance, facial weakness or trauma. Pure tone audiometry was performed in double walled sound proof room using standard procedures. The frequencies tested for air conduction were 125, 250, 500, 1000, 2000, 4000 and 8000 Hz and for bone conduction were 250, 500, 1000, 2000, 4000 Hz. The high resolution computer tomography (HRCT) scan of the temporal bone of proband was performed in axial and coronal planes using 0.7 mm thick section to confirm the audiological findings. Her general health was good. There was no history of hearing impairment in her family. Both parents and other family members were examined. The 59 year old father, 49 year old mother, 23 year old sister, 20 year old brother and other relatives were asymptomatic. Clinically otoscopic findings for all the individuals from the family were normal. Similar test procedure were followed for all other cases and controls included in this study. Informed written consents were obtained from all the individuals participating in this study. This study was approved by Institutional Ethical Committees of Institute of Life Sciences and SCB Medical College and the methods were carried out in accordance with approved guidelines.

### Genotyping of TGFB1 promoter mutation −832G > A

To confirm the diagnosis, DNA was extracted from venous blood samples using rapid-non-enzymatic method^19^. Probands daughter (aged 1.5 years) blood sample was collected on Whatman FTA card and DNA was extracted following the manufacturer’s instructions. PCR based single-strand conformation polymorphism (SSCP) analysis was used to detect the −832G > A mutation. The denatured amplicons were electrophoresed on a 10% native polyacrylamide gel and separated strands were visualized by silver staining. Fragments showing aberrant bands were subjected to direct DNA sequencing for ensuring the absence of any genotyping error.

### TGFB1 Expression Analysis

Total RNA was isolated from whole blood using QIAamp RNA blood mini kit (Qiagen) following the manufacturer instructions. The RNA quality and quantity were assessed using Nanophotometer (Implen). cDNA synthesis was performed with 100 ng of total RNA using the QuantiTect reverse transcription kit (Qiagen). To quantify the total mRNA expression of TGFB1, fluorescence based real time PCR (QRT-PCR) was performed using QuantiTect SYBR Green RT-PCR Kit (Qiagen) with 2 μl of cDNA and 4 pmol of each primer in a total reaction volume of 25 μl. Target sequence of TGFB1 mRNA was amplified using previously described gene specific primers and conditions^3^. The comparative ∆C_T_ method (∆∆C_T_ method) was used to quantity the TGFB1 mRNA level relative to the average expression of the β-actin and 18S rRNA housekeeping genes. The data for each sample and parameters were averaged and relative expression levels were compared using Graph pad Prism software. TGFB1 plasma levels were estimated in triplicate using enzyme linked immunosorbent assay (ELISA) with Human LAP TGFB1 elisa ready-set-go kit.

### Luciferase Reporter Assay

To test whether the TGFB1 promoter variant −832G > A affects gene expression, 1199 bp from wild type and mutated promoter region were subcloned into the SacI and HindIII sites of pGL3 basic promoter less vector (Promega) which contains the firefly luciferase gene (F-luc). TGFB1 promoter plasmids containing firefly luciferase reporter genes (F-luc) were transiently transfected in to human embryonic kidney (HEK) 293 cells plated in 6 well format (1 × 10^4^ cells/well), using Lipofectamine 2000 reagent (Invitrogen). Renilla vector containing the Renilla luciferase gene (R-luc) was cotransfected to serve as an internal control for transfection efficiency. Luciferase assays using the Dual-glo luciferase assay system (Promega) were performed 24 hours after transfection. 50 μl of Dual-glo luciferase reagent was added to the 50 μl of culture medium and incubated at room temperature for 10 minutes. The F-luc luminescence was measured using luminometer (Promega). Before measuring of R-luc, 50 μl of the stop and glo reagent was added to quench the F-luc reaction. The R-luc luminescence was measured after an incubation of 10 minutes at room temperature. Results were expressed as F-luc/R-luc means ± SEM.

### Electrophoretic mobility shift assay (EMSA)

Further, we assessed the functional consequence of the 1-bp substitution by electrophoretic mobility shift assay (EMSA) to see the altered binding of two transcription factors in the mutated promoter sequence. All reactions included double stranded, ^32^P-labeled, oligonucleotides probes corresponding to wild type and mutated TGFB1 promoter nucleotides. EMSA was performed on MCF7 cells nuclear proteins. Nuclear protein extract was incubated with ^32^P-labeled probe in binding buffer at room temperature before loading on to 8% polyacrylamide gel. The samples were then electrophoresed at 160V for 4 hour in cold condition. After migration, gel was dried and exposed overnight at −20 °C. Visualization was carried out using a Kodak infrared Imager system.

## Additional Information

**How to cite this article**: Priyadarshi, S. *et al*. Otosclerosis Associated with a De Novo Mutation −832G >A in the TGFB1 Gene Promoter Causing a Decreased Expression Level. *Sci. Rep.* **6**, 29572; doi: 10.1038/srep29572 (2016).
